# Benzoate of Guaiacol

**Published:** 1903-01

**Authors:** Mark W. Peyser

**Affiliations:** Richmond Va., Assistant Physician to the Home for the Aged and Infirm, Secretary of the Richmond Academy of Medicine and Surgery, Etc.


### BENZOATE OF GUAIACOL.*

BY MARK W. PEYSER, M.D., RICHMOND VA.,

Assistant Physician to the Home for the Aged and Infirm, Secretary of the Richmond Academy of Medicine and Surgery, Etc.

Four years ago I read before the Academy a paper giving a brief account of the properties of the benzoate of guaiacol (sometimes called benzoyl-guaiacol and benzosol), with reports of cases of bronchitis, pneumonia and tuberculosis satisfactorily treated by its means. Since that time, I have employed the remedy in a variety of conditions, the results, though not uniformly successful, being all that one could hope for in such an uncertain practice as medicine.

In a number of respiratory diseases, the salt is one that appears ideal, containing, as it does, both guaiacol and benzoic acid. In cystic affections the acid content is of excellent avail. In both of these classes we have the remote local action of the agent, an action which is too often neglected. The benzoate when swallowed is decomposed in the intestines, chiefly the small, into benzoic acid and guaiacol, and, according to Butler, exerts a favorable influence

*Read before the Richmond Academy of Medicine and Surgery, February 25, 1902.

in intestinal fermentation, auto-intoxication, diarrhea, etc. My experience in this direction has been limited, but agrees, so far, with the statement. I have had no experience with it in the treatment of diabetes, in which disease it is said to have been administered with good results.

There are certain advantages possessed by the benzoate over guaiacol and its carbonate and creosote. It is almost tasteless and odorless, and while during the first few days of its administration there may be some objection, the patient soon becomes accustomed to it and no longer rebels; not so with the other preparations. It contains, when pure, no cresols, which are present even in the purest guaiacol, and, therefore, it does not irritate the stomach nor cause burning and itching of the skin. The dose for an adult is from five to ten grains gradually increased, the maximum per day being forty grains; but the length of time required to produce the maximum effect is shorter than those of the other preparations. The remedy may be administered in capsules, powder or in suspension, either alone or in combination. Below is a further report of cases in which it was given:

Mrs. J. M. D. Cystitis. There had been several acute attacks at longer or shorter intervals, and various remedies had been used with more or less success, none, however, overcoming the inflammatory state entirely. Eight five-grain capsules of the benzoate finally succeeded in doing so, since which time, so far as I know, there has been no other seizure.

C. H. E. Microscopic examination made by Dr. Greer Baughman shows that this patient has myosarcoma of the bladder. When the urine becomes scalding and its passage agonizing, the administration of a few five-grain doses of the benzoate results in speedy relief.

F. W. Prostatitis. The pain and cystitis incident readily succumb to a combination of five grains of benzosol and a fourth grain of codeine given at three-hour intervals.

Mrs. C. L. P. Acute nephritis, first seen in fourth day of illness. Owing to the intense pain in the back and lower abdomen, and whenever urine was voided, the remedy was prescribed in the combination just described. The following day she reported much relief from pain and easy urination. Examination of the urine, three quarts of which she passed in the preceding twenty-four hours, showed 2 per cent. of albumin, a few casts and a large number of kidney cells. The medicine was continued, flannel underwear ordered and a strictly milk diet enjoined. The next day the quantity of urine was two and a half quarts, albumin a little less than 1 per cent., kidney cells were much diminished and casts absent. On the fourth day of treatment the quantity of urine was normal, and there was but a trace of albumin and very few cells. On the fifth day albumin had disappeared entirely. Pain was not a factor in the case after the third day, when the codeine was discontinued. The patient is now taking the ammonio-acetate of iron.

R. D. and A. R. M., each aged two years. Acute bronchitis with temperatures respectively of 101° and 103°. Benzoate of guaiacol suspended in syrup of amorphous quinine was given every three hours. In twenty-four hours all fever, cough and pain had disappeared. This is a fairly common result; indeed, in some cases of acute laryngitis, I have seen the cough cease almost entirely in twelve hours.

Mrs. J. H. Acute bronchitis. A hot mustard foot bath and a mustard plaster to the chest were ordered, and the combination of the benzosol and codeine were prescribed. The next day, pain having ceased, codeine was stopped, but the benzosol was continued for two days longer, when symptoms disappeared.

S. J., Jr. Acute lobar pneumonia. The benzoate was given suspended in syrup of quinine. In five days after the beginning of the disease, and three days after I had first seen him, the crisis ensued. The attack was short, but sharp, and strychnine and atropine were employed coincidently. In one or two other cases, I have seen the crisis appear on the fifth day under the use of the benzoate.

Mrs. T. E. W. An attack of pneumonia and typhoid fever in March, 1899, left this patient frail and weak. The benzoate was given during the course of her illness, with excellent effect, and was continued for some time afterward. I saw no more of the patient professionally, till the 9th of January, this year, when she had a pulmonary hemorrhage. Suprarenal capsules checked this successfully. She was then given the benzoate of guaiacol, the use of which she has continued ever since. A day or two ago, she informed me that she weighed more now than ever before in her life, with the exception of the time she spent in the mountains after her first sickness. This patient is the only one in my experience, who has complained of eructations produced by the remedy.

With this account I shall conclude, desiring to say, however, that there were some cases in which the benzoate of guaiacol was of no avail; but the majority in this report were chosen at random, and from it my hearers may judge of the value of the agent. If they incline to use it, I trust their results will be as satisfactory as mine.

*No. 303 Twelfth Street, North.*

